# On the Pathological Anatomy of Puerperal Fever

**Published:** 1862-08

**Authors:** 


### ON THE PATHOLOGICAL ANATOMY OF PUERPERAL FEVER.

By PROFESSOR BUHL.

Professor Buhl, of Munich, having examined the bodies of fifty women who died of puerperal fever, states that a constant and characteristic appearance is a pappy, red or dark brown or grayish-black mass lining the inner wall of the uterus, giving forth sometimes a gangrenous and sometimes a putrefactive smell. It is this matter which supplies the poisonous infection of puerperal fever; but as to the cause of the production of the fever, differences of opinion prevail; some regarding it as the consequence of the immediate passage of poisonous matter into the womb, while others think that a preliminary poisoning of the blood by misasmata takes place, the corrupted mass being only a secondary result. Anatomically, we may distinguish two forms of puerperal fever—puerperal pyæmia and puerperal peritonitis—forms which may be clinically distinguished, as it is of importance in prognosis that they should be so.

Puerperal pyæmia does not usually prove fatal before the ninth day, and frequently not until after the third week. It is chiefly met with where the disease does not put on an epidemic form, the veins being the channel of infection; coagula, accompanied by suppuration, being found in the veins of the walls of uterus, in a pampiniform plexus or in a spermatic vein. In no instance did the author ever find both spermatic veins obstructed, and in only one case was the entire vena cava inferior filled with adherent coagula. These coagula and their subsequent caseous metamorphosis are quite sufficient to establish the existence of puerperal pyæmia, the so called metastatic abscesses being seldom met with. Œdema of the lungs and ecchymosis of the pleura were frequently met with by the author.

The puerperal peritonitis was more frequent, more violent, and more rapidly fatal than the puerperal pyæmia, inasmuch as death sometimes occurred within two days after delivery, and in but few cases was delayed to the third week. Of the 32 cases of this variety only 2 were chronic, proving fatal in the course of six or eight weeks. In all the cases purulent exudation was found, in 18 instances occupying the tubes, and in 14 the subserous tissue of the uterus. The two conditions were found combined in only 4 instances, and a plugged condition of the veins was observed only in 5 instances. Of the 18 instances in which puerperal pyæmia occurred, in only 2 was there pus in the tubes, and in only 1 subserous effusion of pus; so that of 20 cases of tubal suppuration, in 18 peritonitis was present, and of the 14 cases of subserous suppuration peritonitis occurred in 13. On the other hand, of 23 cases of purulent coagula of the veins, in only 5 did peritonitis occur, and in all these there was subseous or tubal suppuration also, and in 16 cases in which these parts exhibited no pus, no peritonitis took place. The disease of the veins thus bore no relation to the occurrence of peritonitis. It results from these facts, that peritonitis may arise either from the immediate passage of the poisonous material from the uterus through the tubes, or from the conveyance of this from the inner wall of the uterus by the lymphatics. The supposition that the pus may have proceeded from the perritoneum into the tubes is negatived by the fact of these having been free of it in 14 cases; and the pus of the peri-uterine, subserous tissue or of the lymphatic vessels must be regarded rather as a consequence than a cause of the peritonitis, inasmuch as it was absent here in twenty instances. The prognosis is not alike in these two modes of origin of the peritonitis. That induced by the pus from the tubes is a much slighter and more simple inflammatory process, met with when there is little or no epidemic extension of the disease; while the peritonitis resulting from lymphatic absorption is a much severer form of disease, preceding or accompanying general infection, and is especially met with in the epidemic form.

In both of the principal forms of puerperal fever, besides the morbid uterine appearances there were found—1. Almost constantly swelling and watery infiltration of the retro-peritoneal, inguinal, and (though seldomer) the mesenteric glands. 2. Osteophytes on the internal surface of the cranium. 3. In several cases, especially in pyæmia and lymphatic absorption, a distention of the cortical substance of the kidney, together with the microscopical appearances corresponding to the acute stage of Bright's disease. In only two of fifty individuals was tuberculosis found.—*Froriep's Notizen.* 1861. No. 13.—*Med. Times and Gazette.*

